# T86. COGNITIVE SUBTYPES IN FIRST-EPISODE PSYCHOSIS: AN EMPIRICAL LONGITUDINAL STUDY OF RELATIONSHIP TO COGNITIVE, SYMPTOM AND FUNCTIONAL OUTCOMES

**DOI:** 10.1093/schbul/sby016.362

**Published:** 2018-04-01

**Authors:** Andrew Watson, Bill Deakin, John Suckling, Paola Dazzan, Stephen Lawrie, Rachel Upthegrove, Nusrat Husain, Imran Chaudhry, Graham Dunn, Peter Jones, Danuta Lisiecka, Shon Lewis, Thomas Barnes, Stephen Williams, Stephen Hopkins, Emma Knox, Kelly Byrne, Richard Drake, Richard Smallman, Eileen Joyce

**Affiliations:** 1 UCL Institute of Neurology; 2 University of Manchester; 3 University of Cambridge; 4 Institute of Psychiatry, Psychology and Neuroscience, King’s College London; 5 University of Edinburgh; 6 University of Birmingham; 7 Imperial College London

## Abstract

**Background:**

Variable outcomes following a first-episode of psychosis are partly attributable to heterogeneity in cognitive functioning. Previous work in first episode psychosis has identified clinically meaningful cognitive sub-types based on pre-specified differences in estimated premorbid and current cognitive functioning. We used an empirical clustering technique to examine whether these cognitive profiles can be replicated with an unbiased method, their relationship with clinical, cognitive and global functioning at psychosis onset as well as their stability over time.

**Methods:**

Patients attending NHS early intervention services following a first episode of psychosis were recruited to a double-blind clinical trial of minocycline for negative symptoms of psychosis (BeneMin) which found no treatment effect. Participants were assessed on clinical, cognitive and global functioning at baseline (n=169) and 12-month follow-up (n=107). K-means analysis was used to empirically cluster participants on the basis of estimated premorbid IQ (WTAR), and baseline cognitive functioning (derived from 4 sub-tests of the WAIS IV, verbal fluency (COWAT) and verbal learning scores (AVLT)). Clinical and global functioning was assessed using the Positive and Negative Syndrome Scale (PANSS), Calgary Depression Scale (CDS) and Global Assessment of Functioning (GAF).

**Results:**

K-means cluster analysis revealed three cognitive subgroups: 28% showing preserved premorbid and current IQ (PIQ); 29% displaying compromised premorbid and current IQ (CIQ); and 43% with normal premorbid but deteriorated current IQ (DIQ). There were no significant differences between groups in age, gender, CDS, cannabis use or olanzapine equivalent antipsychotic dose. The PIQ group performed significantly better than the DIQ group on all baseline cognitive measures, who performed significantly better than the CIQ group. At baseline, there were significant differences in GAF scores (F(2,168) = 4.267, p=0.016; PIQ >DIQ, LIQ). At 12-months, all three groups had improved over time on cognitive function with no group x time interactions except verbal fluency (F(2, 97) = 5.204,p=0.007) where only the DIQ group improved. All groups improved on positive and total symptom scores but only the PIQ and DIQ groups significantly improved on negative and general symptom scales. There was no significant change in GAF score in any of the groups over time. At follow-up there were significant differences between groups in negative syndrome scores (F(2, 104) = 8.720, p=0.004: LIQ > PIQ, DIQ) and global functioning (F(2,101) = 5.880 p=0.004; PIQ > DIQ, LIQ).

**Discussion:**

Using an unbiased method to define cognitive subgroups at first episode, we confirmed in a new sample, previous findings which used pre-specified criteria. A large subgroup showed evidence of a decline in IQ at psychosis onset and 12-months later this subgroup had neither continued to deteriorate nor returned to premorbid levels of cognition. Patients with preserved normal and compromised cognitive function at psychosis onset showed no deterioration over 12 months. The cognitive sub-groupings were clinically meaningful. The preserved group showed better general function which persisted over 12 months. General functional outcome in the IQ decline group was as poor as the compromised group and the compromised group had more persistent negative symptoms.

